# The effects of the court-type Thai traditional massage on anatomical relations, blood flow, and skin temperature of the neck, shoulder, and arm

**DOI:** 10.1186/s12906-016-1282-y

**Published:** 2016-09-15

**Authors:** Vasana Plakornkul, Manmas Vannabhum, Yadaridee Viravud, Jantima Roongruangchai, Pramook Mutirangura, Pravit Akarasereenont, Tawee Laohapand

**Affiliations:** 1 Department of Anatomy, Faculty of Medicine Siriraj Hospital, Mahidol University, Bangkok, Thailand; 2 Center of Applied Thai Traditional Medicine, Faculty of Medicine Siriraj Hospital, Mahidol University, Bangkok, Thailand; 3 Department of Vascular Surgery, Faculty of Medicine Siriraj Hospital, Mahidol University, Bangkok, Thailand

**Keywords:** Massage, Anatomy, Blood flow, Skin temperature, Neck, Arm

## Abstract

**Background:**

Court-type Thai traditional massage (CTTM) has specific major signal points (MaSP) for treating musculoskeletal conditions. The objectives of this study are to investigate the anatomical surfaces and structures of MaSPs, and to examine blood flow (BF) and skin temperature (ST) changes after applying pressure on the MaSPs on neck, shoulder, and arm areas.

**Methods:**

In the anatomical study, 83 cadavers were dissected and the anatomical surfaces and structures of the 15 MaSPs recorded. In human volunteers, BF, peak systolic velocity (PS), diameter of artery (DA), and ST changes were measured at baseline and after pressure application at 0, 30, 60, 180, and 300 s.

**Results:**

There was no statistical difference in anatomical surfaces and structures of MaSP between the left and right side of the body. The 3 MaSPs on the neck were shown to be anatomically separated from the location of the common carotid arteries. The BF of MaSPs of the neck significantly and immediately increased after pressure application for 30 s and for 60 s in the arm (*p* < 0.001). ST increased significantly and immediately after pressure application for 300 s (*p* < 0.001). There was no significant correlation between BF and ST at any of the MaSPs.

**Conclusions:**

This study showed that MaSP massages were mainly directed towards muscles. MaSPs can cause significant, but brief, increases in BF and ST. Further studies are suggested to identify changes in BF and ST for all of the MaSPs after actual massage treatment sessions as well as other physiological effects of massage.

## Background

Court-type Thai traditional massage (CTTM) - *Raja Sum Nak* in Thai - is a practice of Thai traditional medicine. CTTM is considered an alternative or adjunctive treatment for people with musculoskeletal symptoms that are widely recognized in Thailand and worldwide [1]. A randomized controlled trial was demonstrated that the administration of CTTM twice a week for 4 weeks decreases pain intensity and significantly increases pressure-pain threshold compared to amitriptyline in patients with chronic tension-type headaches [2]. CTTM also helps rehabilitate physical fitness in patients suffering from diseases such as paralysis. A previous randomized controlled trial showed that 6 weeks of CTTM results in similar outcomes to physical therapy program in elderly stroke patients, including decreased spasticity, increased functional ability, and improved quality of life [3]. Moreover, CTTM group also had decreased anxiety and depression scores in elderly stroke patients when compared to the physical therapy group [3].

CTTM has one unique characteristic which sets it apart from other types of massage such as deep tissue massage, friction massage and trigger point massage. In CTTM, the practitioner always uses only their thumbs, fingers or palm to gently exert pressure [1, 4]. CTTM practitioners use their thumbs for 30 to 45 s to deliver pressure through specific points which are called major signal points (MaSP), *Jude San Yan Mae* in Thai. There are 50 MaSP points along the whole body [1, 5]. These MaSPs are tacit knowledge which has been passed from generation to generation. In Thai traditional massage books [1, 4], the MaSPs which are being used now are taught by Narongsak Boonratanahiran, a master practitioner of CTTM who had been taught by CTTM practitioners in the Royal Palace. As it has been taught, MaSPs are believed to correlate to locations of anatomical structures. CTTM practitioners use MaSPs for both diagnosing and treating specific conditions.

While other types of massages demonstrate several physiological and neurological effects [6–11], evidence is still lacking for CTTM. The only published evidence is a descriptive study of CTTM administered to the lower extremities which reports that a 15-min massage to the right leg is associated with an increase in temperature on the dorsum of both feet, as well as an increase in pulse rate and a decrease in systolic blood pressure [12].

This study focuses on the neck, shoulder, and arm areas. Musculoskeletal pain in these areas are most common among patients who work using computers and office workers [13]. The first objective of this study is to investigate the anatomical surface landmarks of MaSPs of the shoulder, medial side of the arm, and lateral side of the arm and the anatomical structures below. The second objective of this study is to examine the physiological effects of applying pressure on the MaSPs of the shoulder, medial side of the arm, and lateral side of the arm on BF and ST. We hypothesize that, as is traditionally believed, MaSPs are actually correlated to the location of specific nerves, blood vessels, and muscles in these underlying areas. The pressure applied is associated with increase in BF and ST to those areas.

## Methods

### Experimental design

This descriptive study was divided into 2 parts: (1) anatomical study and (2) human study. The protocol was approved by the committee of Siriraj Institutional Review Board, Faculty of Medicine Siriraj hospital, Mahidol University, Bangkok, Thailand.

### Anatomical study

#### Cadaver

The necks and arms of 81 embalmed cadavers with a total of 162 sides were used: 51 males and 30 females, aged between 33 and 99 years and mean aged 69.6 ± 15.3 years (mean ± SD). The inclusion criteria for cadavers were all cadavers who signed body donation consent prior to their deaths to Department of Anatomy, Faculty of Medicine Siriraj Hospital, Mahidol University, Thailand, and were kept in supine position in the anatomical laboratory during 2012. Cadavers with history of bone fractures, abnormalities, tumor, trauma, skin lesion, and surgical lesion involving the neck, shoulders, and arms were excluded from this study.

#### Dissection

**Fig. 1:**
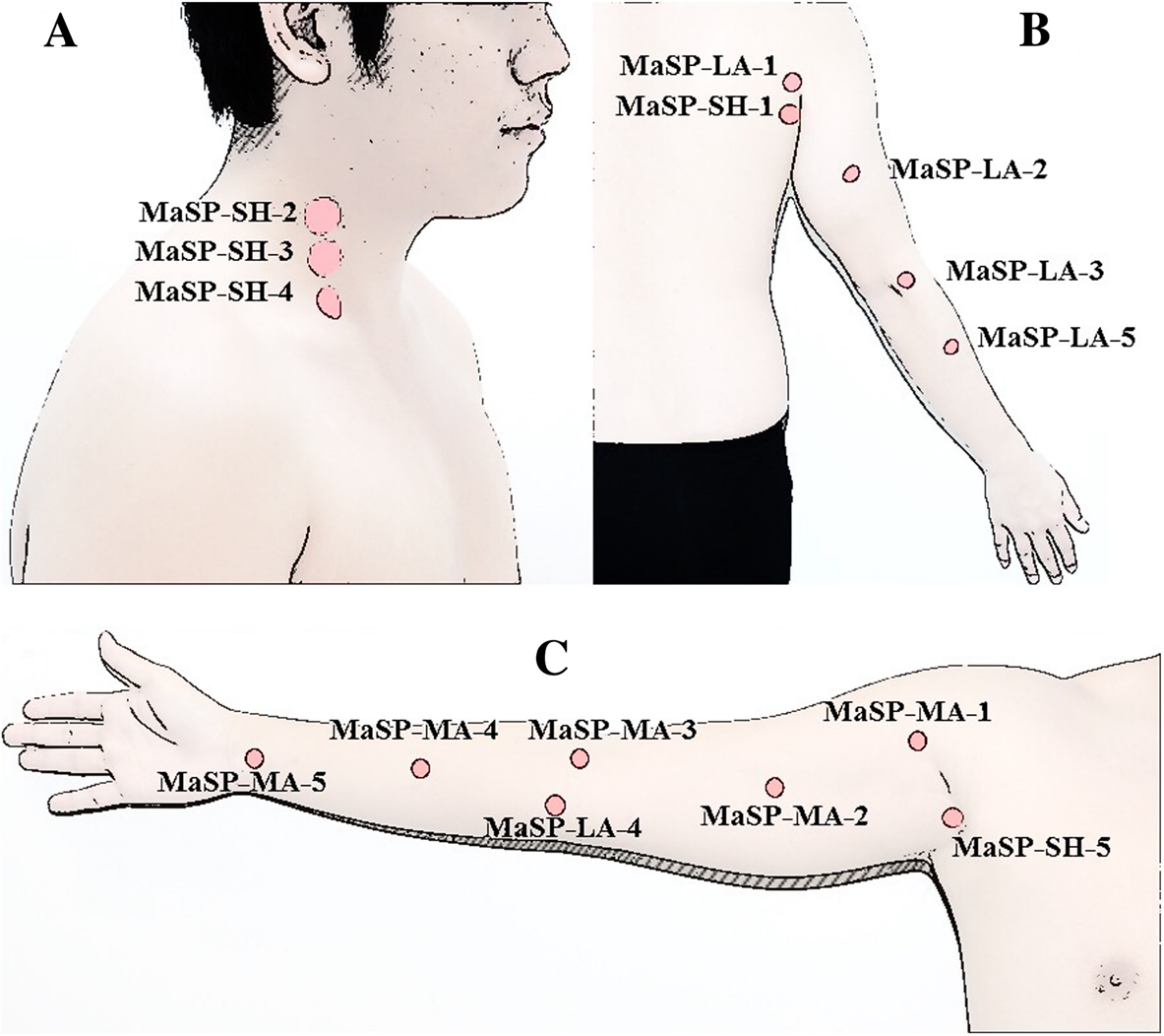
Fifteen MaSPs of the neck, shoulder, and arm were divided by location: 5 points on neck & shoulder (MaSP-SH-1 to 5), 5 points on medial side of the arm (MaSP-MA-1 to 5) and 5 points on lateral side of the arm (MaSP-LA-1 to 5). **a**, **b** and **c** showed *right *lateral of the neck view, posterior of the arm view, and anteromedial of the arm view, respectively

The study explored 15 MaSPs as detailed in Table 1 and Fig. 1. All cadavers were marked on each side with pins by three experts in CTTM from the Center of Applied Thai Traditional Medicine, Faculty of Medicine Siriraj Hospital, Mahidol University, Thailand who have been in practice for more than 15 years. The surface landmarks of each point were measured after marked pins. In order to determine the anatomical area for an exploratory dissection, acrylic color was injected at each marked MaSPs using 25 cc syringes and number 18 needles. Importantly, all points were injected with the same direction and angle as the force applied during CTTM practice. Afterwards, the pins were removed and the cadavers were dissected from superficially. Single-thumb MaSP and double-thumb MaSP, presence of color within a 1-inch and 1.5-inch circular area marked area for exploratory dissection, respectively (Table 1). The color marker falls outside this circular area were not defined as anatomical structures of the MASPs. After the exploratory dissections were performed, data regarding the presence of nerve, blood vessels, muscles, and bone were collected.

**Table 1:** Fifteen MaSPs are investigated in this study

Area	MaSPs	Massage-thumb	Figure
Neck	MaSP-SH-2	single-thumb	1A
	MaSP-SH-3	single-thumb	1A
	MaSP-SH-4	single-thumb	1A
Shoulder	MaSP-SH-1	single-thumb	1B
	MaSP-LA-1	single-thumb	1B
	MaSP-SH-5	single-thumb	1C
Arm	MaSP-MA-1	single-thumb	1C
	MaSP-MA-2	double-thumb	1C
	MaSP-MA-3	double-thumb	1C
	MaSP-MA-4	double-thumb	1C
	MaSP-MA-5	double-thumb	1C
	MaSP-LA-2	single-thumb	1B
	MaSP-LA-3	double-thumb	1B
	MaSP-LA-4	double-thumb	1C
	MaSP-LA-5	double-thumb	1B

### Human study design

#### Participants

Thirty healthy volunteers (15 male and 15 female) who met the following inclusion criteria: age 18 to 45, Body Mass Index (BMI) 18 to 25 kg/m^2^, had normal vascular structures of neck and arm (both artery and vein) on right side of the body, confirmed by Duplex ultrasound. Subjects were excluded if the participant: had one arm, was an athlete or a person who exercised more than 5 times per week, smoking, alcoholism, trauma, bone fracture, inflammatory joint diseases (arthritis, rheumatoid, gout), skin diseases, infectious diseases (tuberculosis or AIDs), history of operation, had musculoskeletal symptoms, neurological, cardiovascular diseases, hypertension, DM, fever (>38.5 °C), menstruation period, and pregnancy at time of recruitment. Participants willingly joined this study and signed informed consent prior to enrollment. Consent to publish the images in Figs. 1 and 5 were obtained from both of the participants featured.

#### Intervention

The BF and ST studies were done in separate sessions. All sessions were conducted at the same time each day to reduce physiological changes that might occur from the effects of circadian rhythms [14]. At least 4 h before the study, participants were told to refrain from exercise. At least 2 h, they were asked to avoid to extremely hot and cold environments, as well as refrain from caffeine-containing food and beverages. Male participants were asked to take off their shirt, while female participants were given vests which allowed access to the neck, shoulder, and arm. Ambient room temperature was controlled for 22–24 °C, without air flow directly to participants throughout the study. Participants were given a 15-minute rest period before vital signs were recorded. One CTTM expert from the cadaver-session performed all the MaSP massages.

#### Blood flow measurement

**Fig. 2:**
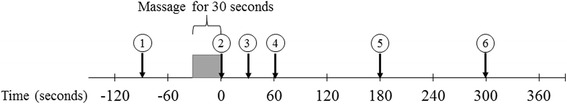
Experiment procedure of this study. 1) baseline (before pressure 60 s), 2) after pressure application at 0 s, 3) after pressure application at 30 s, 4)) after pressure application at 60 s, 5) after pressure application at 180 s, and 6) after pressure application at 300 s

The BF studies were done at the Vascular Surgery clinic, Siriraj Hospital, Mahidol University, Bangkok, Thailand. BF measurement via the LOGIQ E9 (GE Healthcare, Milwaukee, Wis., USA) with probes 9L - RS (3.33–10 MHz). Sixty seconds before applying pressure to each MaSP, BF, PS, and DA on the right arm were recorded as baseline. After application of water-soluble gel, duplex ultrasound probes were placed directly adjacent to the 7 MaSPs. Vessel locations were confirmed by spectral image and audio signals produced by Duplex ultrasound. BF associated with MaSP-SH-2 to 4 were measured using the right common carotid artery and probe was placed at 1.5 to 2 cm proximal to its bifurcation. BF associated with MaSP-MA-2 was measured using the right brachial artery, and the probe was placed at bicipital furrow of the medial side of the arm, 5 cm proximal to the elbow. BF associated with MaSP-MA-3 and 4 were measured using the right radial artery, and the probe was placed on the lateral side of the wrist, 1 to 2 cm proximal to the wrist. Finally, BF associated with MaSP-MA-5 was measured using the right radial artery, and the probe was placed on the radial side of the dorsum of the hand, 1 to 2 cm distal to the wrist. Each MaSP was pressed for 30 s while the probe was fixed over the artery. BF was recorded immediately after each release of MaSP at 0, 30, 60, 180, and 300 s (Fig. 2). After a 5-minute rest period, participants filled out a questionnaire regarding their perceived sensation during MaSP administration. BF, PS, and DA were analyzed, and flow volume measurements were repeated twice for minor errors such as angle correction and diameter measurement.

#### Skin temperature measured with thermographic camera

The ST studies were done at the Ayurved School, Center of Applied Thai Traditional Medicine, Faculty of Medicine Siriraj Hospital, Mahidol University, Bangkok, Thailand. The environmental conditions remained the same as BF session. Importantly, the room and the participants are illuminated only with indirect lighting sources. During this session, participants were asked to avoid right side of the arm and neck contact with things in order to avoid transferring the heat which can interfere with the accuracy of ST. Participants were asked to remain in a fixed cross-legged sitting position on the floor. The right arm abducted at 90 degree and the edge of the right hand placed on the chair seat. Sixty seconds before applying pressure to each MaSP, ST was recorded by infrared thermographic camera (TVS-500) as baseline. Then, ST was recorded immediately after each release of MaSP at 0, 30, 60, 180, and 300 s (Fig. 2). After a 5-minute rest period, participants filled out a questionnaire regarding their perceived sensation during MaSP administration.

The infrared thermographic camera recorded average the skin temperature over a specified area from both the anterior and posterior aspects of the participants by ThermoController program (version 1.1). Images were selected according to the location MaSPs. The average temperature of each MaSP was compared to other measurement points located along the proximal and distal axis of the MaSP. The anterior MaSPs had six other measurement points while the posterior had 4. The average temperature for all points were measured using Thermography Studio 2007 program (version 4.8).

### Sample size calculation

In the cadaveric study, the sample size was not calculated. The study utilized all cadavers in the Anatomical Laboratory of the Department of Anatomy, Faculty of Medicine Siriraj Hospital, Mahidol University, Thailand. Therefore, 81 cadavers were included in this study. In human study, the estimate sample size was based on our pilot study with the mean of ST increase of 0.9 °C compared to baseline, and a standard deviation of 1.5 with 5 % type I error (2-sided test), power of 90 %, and using the formula $\mathrm{n} = {\left({\mathrm{z}}_{1\hbox{-} \frac{\alpha }{2}} + {\mathrm{z}}_{1\hbox{-} \upbeta}\right)}^2{\upsigma}^2/{\left(\upmu \hbox{-} {\upmu}_0\right)}^2$. Therefore, 30 participants were included in this study.

### Statistical analysis

All analysis were performed using PASW statistics 18.0 (SPSS Inc., Chicago, IA, USA). The descriptive statistic was used for demographic data, distance of surface marking, and questionnaires as mean and standard deviation. The differences in BF, PS, DA, and ST which were computed between baseline (60 s prior to initiation of pressure) and after pressure application in 5 timepoints at 0, 30, 60, 180, and 300 s, were expressed as Mean ± SEM. Multivariate analysis was employed to determine the effect of MaSPs and measurement points over time on BF and ST. Multiple comparisons of differences in temperature between 2 areas at each timepoint, type I error was adjusted by Bonferroni’s methods. The scatter plots were applied to identify the relationship between BF and ST after pressure application. Statistically significant was considered when *p*-value less than 0.05.

## Results

### Anatomical surface landmarks

The anatomical surface landmarks of MaSPs on the neck, shoulder, and arm on cadavers are found as described in Table 2. Both left and right side showed no statistical difference in surface landmarks of MaSP.

**Table 2:** The surface landmarks and anatomical structures deep to the 15 MaSPs

		Vertical of line		Horizontal line		Muscles	Nerves	Vessels
	MaSP	Surface landmarks	Mean length (cm)	Surface landmarks	Mean length (cm)			
1	SH-1	Downward from the top of acromion of scapula	12.8 ± 1.3	Medially from line of spinous process of vertebra	14.5 ± 1.8	• M. Infraspinatus (s) • M. Teres minor (s) • M. Teres major (s)	**-**	• Circumflex scapular artery (d)
2	SH-2	Upward from upper border of clavicle	7.7 ± 1.0	Laterally from body midline	11.5 ± 1.3	• M. Platysma (s) • M. Sternocleidomastoid (m) • M. Levator scapulae (d) • M. Scalenus posterior (d) • M. Scalenus medius (d)	• Cervical plexus (m)	• External jugular vein (s)
3	SH-3	Upward from upper border of clavicle	6.6 ± 0.9	Laterally from body midline	10.2 ± 1.3	• M. Platysma (s) • M. Sternocleidomastoid (m) • M. Levator scapulae (d) • M. Scalenus posterior (d) • M. Scalenus medius (d)	• Cervical plexus (m)	• External jugular vein (s)
4	SH-4	Upward from upper border of clavicle	1.6 ± 0.4	Laterally from body midline	7.5 ± 0.9	• M. Platysma (s) • M. Sternocleidomastoid (s) • Inferior belly of M. Omohyoid (m) • M. Scalenus medius (d) • M. Scalenus anterior (d)	• Brachial plexus (d)	• The 3rd part of subclavian artery (m)
5	SH-5	Posteriorly from the anterior axillary fold	7.8 ± 0.9	Midaxillary line	0	• M. Latissimus dorsi (s) • M. Teres major (s) • M. Subscapularis (m)	• Thoracodorsal nerve (s) • Axillary nerve (d) • Radial nerve (d)	• Thoracodorsal artery & vein (s) • Anterior & posterior circumflex humeral arteries (d) • Circumflex scapular artery (d)
6	MA-1	Downward from the greater tuberosity of humerus	9.1 ± 1.3	Laterally from the anterior axillary fold	2.8 ± 0.9	• M. Biceps brachii (short head) (s) • M. Coracobrachialis (s) • M. Biceps brachii (long head) (d)	• Median nerve (s)	• Brachial artery & vein (s)
7	MA-2	Downward from the greater tuberosity of humerus	15.4 ± 1.4	Medially from medial bicipital furrow	1.8 ± 0.5	• M. Biceps brachii (long head) (s) • M. Coracobrachialis (s) • M. Brachialis (d)	• Median nerve (s) • Ulnar nerve (s)	• Basilic vein (s) • Brachial artery & vein (s)
8	MA-3	Downward from the medial cubital fossa	0	Laterally from the middle point of medial cubital fossa	0	• Biceps brachii tendon (m) • Bicepital aponeurosis (m) • M. Brachialis (d)	• Median nerve (m)	• Cephalic vein (s) • Medial cubital vein (s) • Basilic vein (s) • Brachial artery & vein (m)
9	MA-4	Downward from the medial cubital fossa	11.4 ± 1.0	Laterally from the middle point of medial cubital fossa	2.5 ± 0.8	• Pronator teres tendon (s) • M. Flexor carpi radialis (s) • Palmaris longus tendon (s) • M. Flexor digitorum superficialis (s) • M. Flexor pollicis longus (d) • M. Flexor digitorum profundus (d)	• Median nerve (s) • Ulnar nerve (s) • Anterior interosseous nerve (d)	• Ulnar artery & vein (s) • Radial artery (s) • Anterior interosseous artery & vein (d)
10	MA-5	Downward from the medial cubital fossa	22.8 ± 1.5	Laterally from the middle point of imaginary line was drawn from radial styloid process to ulnar styloid process	0	• Flexor carpi radialis tendon (s) • Palmaris longus tendon (s) • Flexor digitorum superficialis tendon (s) • Flexor carpi ulnaris tendon (s) • M. Pronator quadratus (m)	• Median nerve (s) • Ulnar nerve (s) • Anterior interosseous nerve (d)	• Radial artery (s) • Ulnar artery & vein (s) • Anterior interosseous artery & vein (d)
11	LA-1	Downward from the top of acromion of scapula	10.6 ± 1.2	Medially from line of spinous process of vertebra	12.4 ± 3.5	• M. Deltoid (s) • M. Teres minor (s) • M. Infraspinatus (m)	**-**	• Suprascapular artery and vein (d)
12	LA-2	Downward from the top of acromion of scapula	19.5 ± 2.1	Laterally from the imaginary line was drawn from greater tuberosity of humerus to lateral epicondyle	1.9 ± 0.8	• M. Triceps brachii (lateral head and long head) (s)	• Radial nerve (main branch) (d)	• Radial collateral artery and vein (d)
13	LA-3	Downward from the medial cubital fossa	0	Medially from the lateral epicondyle	2.3 ± 0.5	• M. Brachiolradialis (s) • M. Extensor carpi radialis longus (s) • M. Extensor carpi radialis bravis (m) • M. Brachialis (d)	• Radial nerve (d)	• Radial recurrent artery & vein (d)
14	LA-4	Downward from the medial cubital fossa	0.01 ± 0.2	Laterally from the medial epicondyle	2.1 ± 0.6	• M. Pronator teres (s) • M. Flexor carpi radialis (s) • M. Palmaris longus (s) • M. Flexor carpi ulnaris (s) • M. Flexor digitorum superficialis (d)	**-**	**-**
15	LA-5	Downward from the lateral lepicondyle	9.5 ± 0.9	Laterally from the imaginary line was drawn from lateral epicondyle to ulnar styloid process	2.5 ± 0.6	• M. Extensor carpi radialis bravis (s) • M. Extensor digitorum (s) • M. Extensor digiti minimi (s) • M. Supinator (d) • M. Abductor pollicis longus (d)	• Posterior interosseous nerve (d)	• Posterior interosseous artery & vein (d)

Mean length is mean between left and right sides. No differences between left and right sides (*p* > 0.05)

Abbreviations: *s* superficial layer, *m* middle layer, *d* deep layer

### Anatomical structures deep to the MaSPs

**Fig. 3:**
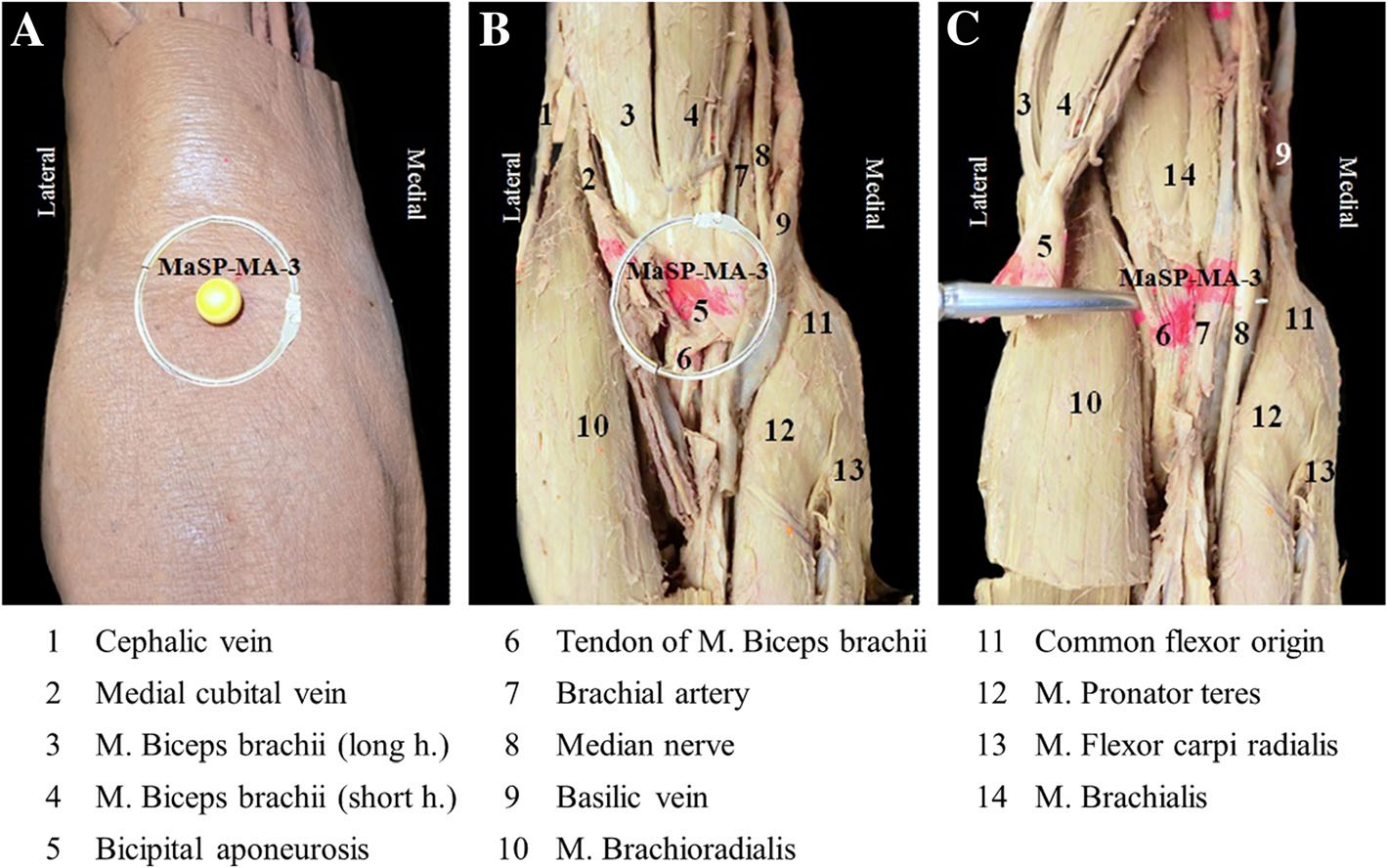
The visual example of the anatomical structures as marked by the presence of the acrylic color inject at the MaSP-MA-3, with its corresponding structures in the circular area. The anterior of the *right elbow* shows anatomical structures from superficial to deep layer. **a** The 1-inch circular area determines the anatomical structures of point. **b** Structures deep to point on superficial layer after removed skin and superficial fascia. **c** Structures in deep layer after flapping M. biceps brachii and aponeurosis

The anatomical structures were marked by the presence of the acrylic color inject at the MaSP, with its corresponding structures in the circular area (Fig. 3). Table 2 shows the description of the anatomical surface landmark and its underlying anatomical structures. In this study, the differences among cadavers are not found. The MaSPs near the right and left common carotid arteries were marked for their distances away from these vascular structures as show in Table 3.

**Table 3:** The distance between 3 of MaSPs of the neck region and the common carotid arteries

MaSP	Mean ± SEM of distance laterally to common carotid arteries (cm)	
	Left	Right
SH-2	3.34 ± 0.05	3.33 ± 0.05
SH-3	3.16 ± 0.04	3.14 ± 0.05
SH-4	4.38 ± 0.06	4.36 ± 0.06

### Characteristics of participants

The general characteristics and vital signs of the participants are shown in Table 4. The participants showed the body temperature, pulse rate, respiratory rate, and blood pressure in normal range during this study.

**Table 4:** Characteristics of participants (*N* =30)

Characteristics	Mean ± SD
Gender	
Male	15
Female	15
Age (mean ± SD, min, max)	21.9 ± 1.8, 19, 25
Male	21.6 ± 1.6, 19, 25
Female	22.6 ± 1.7, 21, 25
Weight (kg)	55.1 ± 7.9
Height (cm)	164.5 ± 7.8
BMI (kg/m^2^)	20.3 ± 1.6
Body temperature before test: axilla (°C)	
Blood flow test	36.7 ± 0.3
Thermal test	36.7 ± 0.4
Pulse rate before test (BPM)	
Blood flow test	79.6 ± 7.2
Thermal test	78.9 ± 7.1
Respiratory rate before test (BPM)	
Blood flow test	15.8 ± 0.8
Thermal test	15.9 ± 1.1
Systolic blood pressure before test (mmHg)	
Blood flow test	107.5 ± 10.6
Thermal test	109.0 ± 8.5
Diastolic blood pressure before test (mmHg)	
Blood flow test	70.3 ± 8.1
Thermal test	71.2 ± 6.9

### Effects of massage on blood flow

**Fig. 4:**
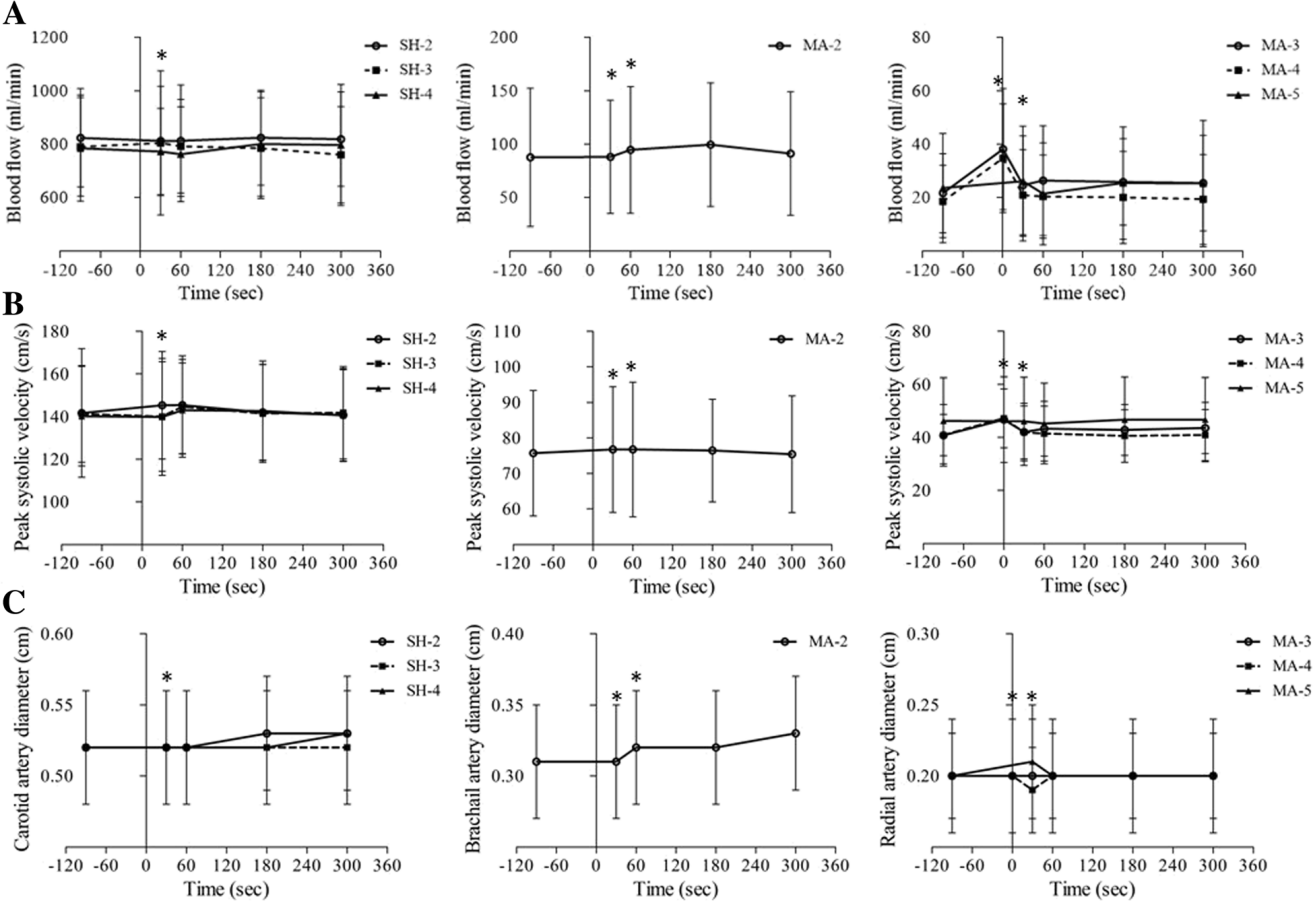
**a** represents comparison in BF, **b** represents PS, and **c** represents DA over time. The *bars* indicate means and standard error of mean (SEM). *p-value < 0.001 for comparison differences between before and after pressure application

BF, PS, and DA changes after pressure application immediately for 30 s. BF, PS, and DA of the right common carotid artery, right brachial artery, and right radial artery show significant differences over time (*p*-value <0.001). BF, PS, and DA of the right common carotid artery measured at MaSP-SH-2 to 4 show statistically significant differences from baseline at 30 s after pressure application (*p*-value <0.001). BF, PS, and DA of the right brachial artery measured at MaSP-MA-2 shows statistically significant differences from baseline at 30 and 60 s after pressure application (*p*-value <0.001). BF, PS, and DA of the right radial artery measured at MaSP-MA-3 to 5 show statistically significant differences from baseline at 0 and 30 s after pressure application (*p*-value <0.001) as show in Fig. 4.

### Effects of massage on skin temperature

**Fig. 5:**
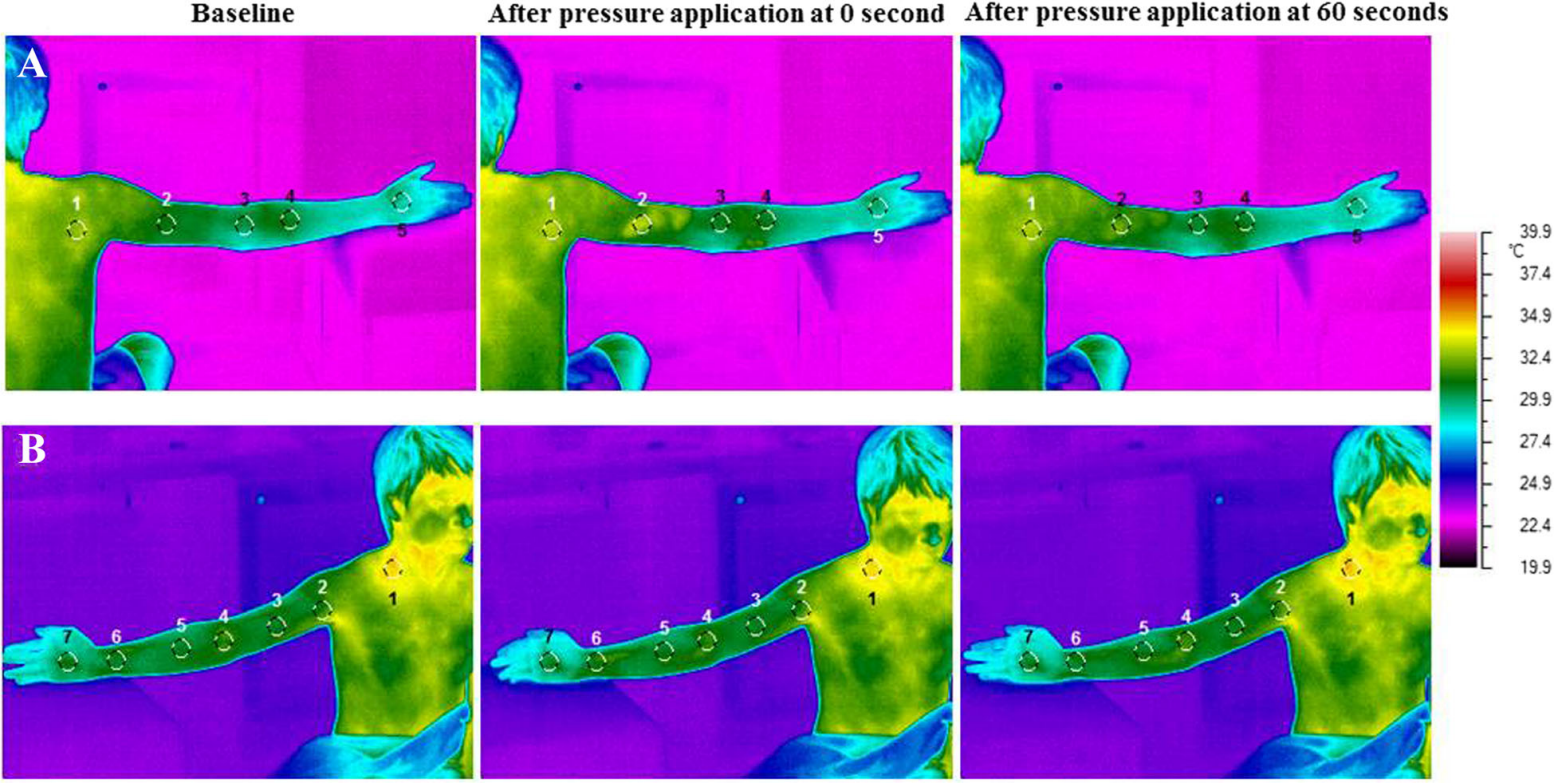
The alteration of ST before and after pressure application at different MaSPs. **a** represents pressure application at MaSP-LA-2 or area 2. This posterior thermographic image view demonstrates 4 other measurement points proximal and distal to the MaSP. Note the color change in area 2 after pressure application. **b** Press at MaSP-SH-4 or area 1, thermographic images were analyzed seven areas from proximal to distal area of posterior image view. Area 1, color was changed to light color after press that represented increasing of ST in that area. Changes in color denote changes in temperature according to the color scale below

**Fig. 6:**
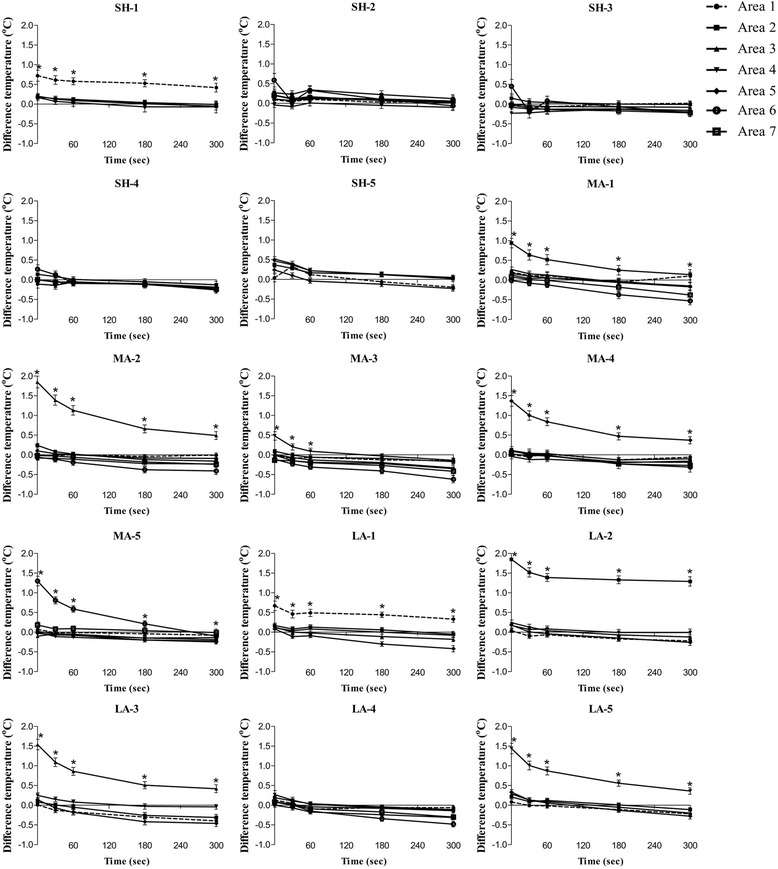
Differences in ST between before and after pressure application of 15 MaSPs, the timepoints were adjusted by baseline (−60 s before press) and presented in the difference of each timepoint after pressure application at 0, 30, 60, 180, and 300 s, respectively. *p-value < 0.05 for comparison between the press area and other areas at every time of the measurement

The alterations of the ST at baseline and after pressure application of each area are shown in Fig. 5. The results of the ST change in all 15 MaSP areas are presented in Fig. 6. There are nine MaSPs which show significant elevation of ST compared to the surrounding measurements points over the entire 300 s (*p*-value < 0.05): These are MaSP-SH-1, MaSP-MA-1, MaSP-MA-2, MaSP-MA-4, MaSP-MA-5, MaSP-LA-1, MaSP-LA-2, MaSP-LA-3, and MaSP-LA-5. MaSP-MA-3 show significant differences in temperature in only at 0, 30, and 60 s.

### Relationship between blood flow and skin temperature

**Fig. 7:**
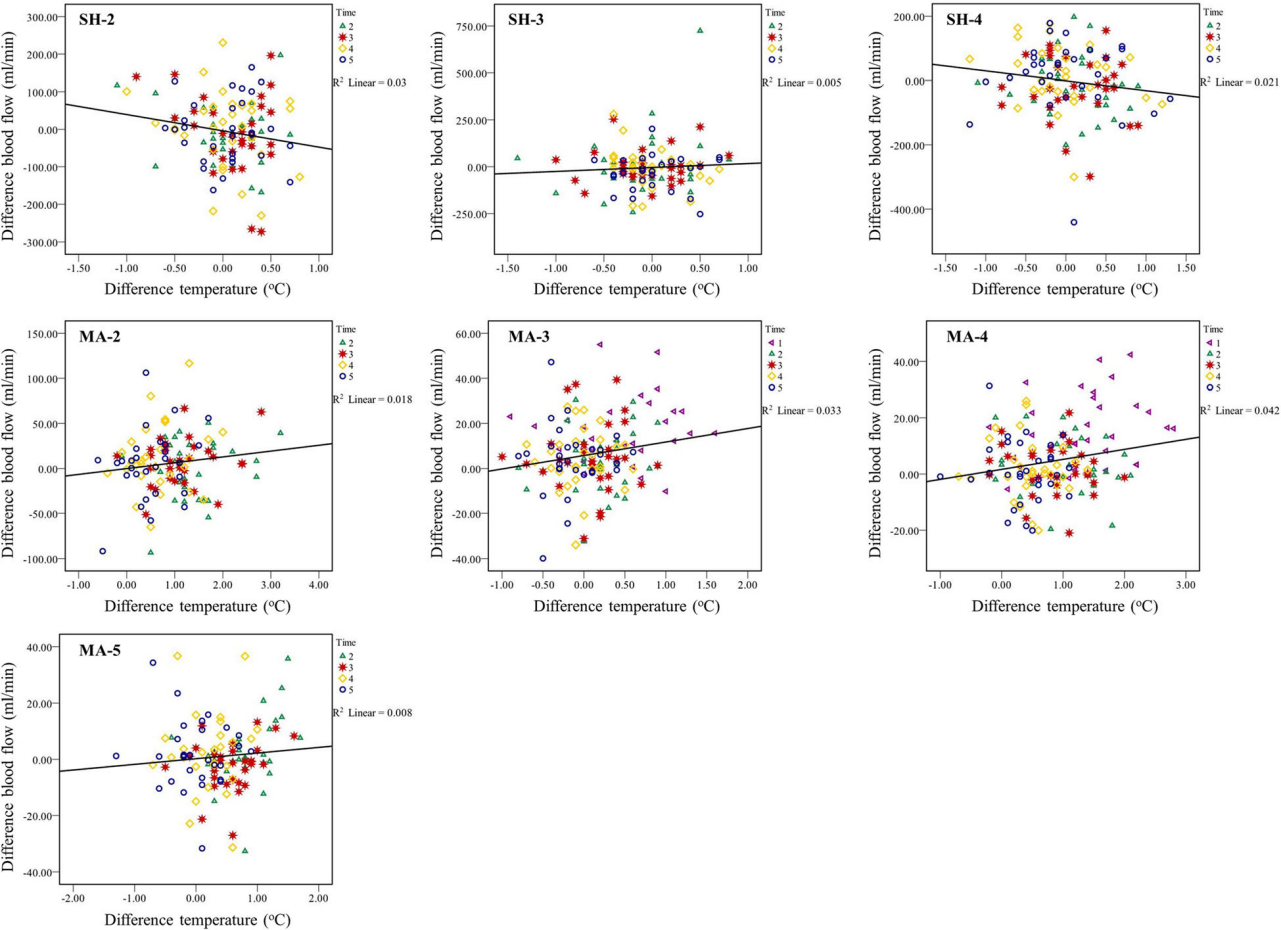
Relationship between differences BF and ST after pressure application of each timepoint: time 1 = 0 s, 2 = 30 s, 3 = 60 s, 4 = 180 s, and 5 = 300 s

Figure 7 shows the relationship between the changes in BF and ST after pressure application at each timepoint: 0, 30, 60, 180, and 300 s of 7 MaSPs. The correlations for each region are as follow. There are no significant correlation between BF and ST at any of the MaSPs.

### The participant perception after MaSP administration

**Fig. 8:**
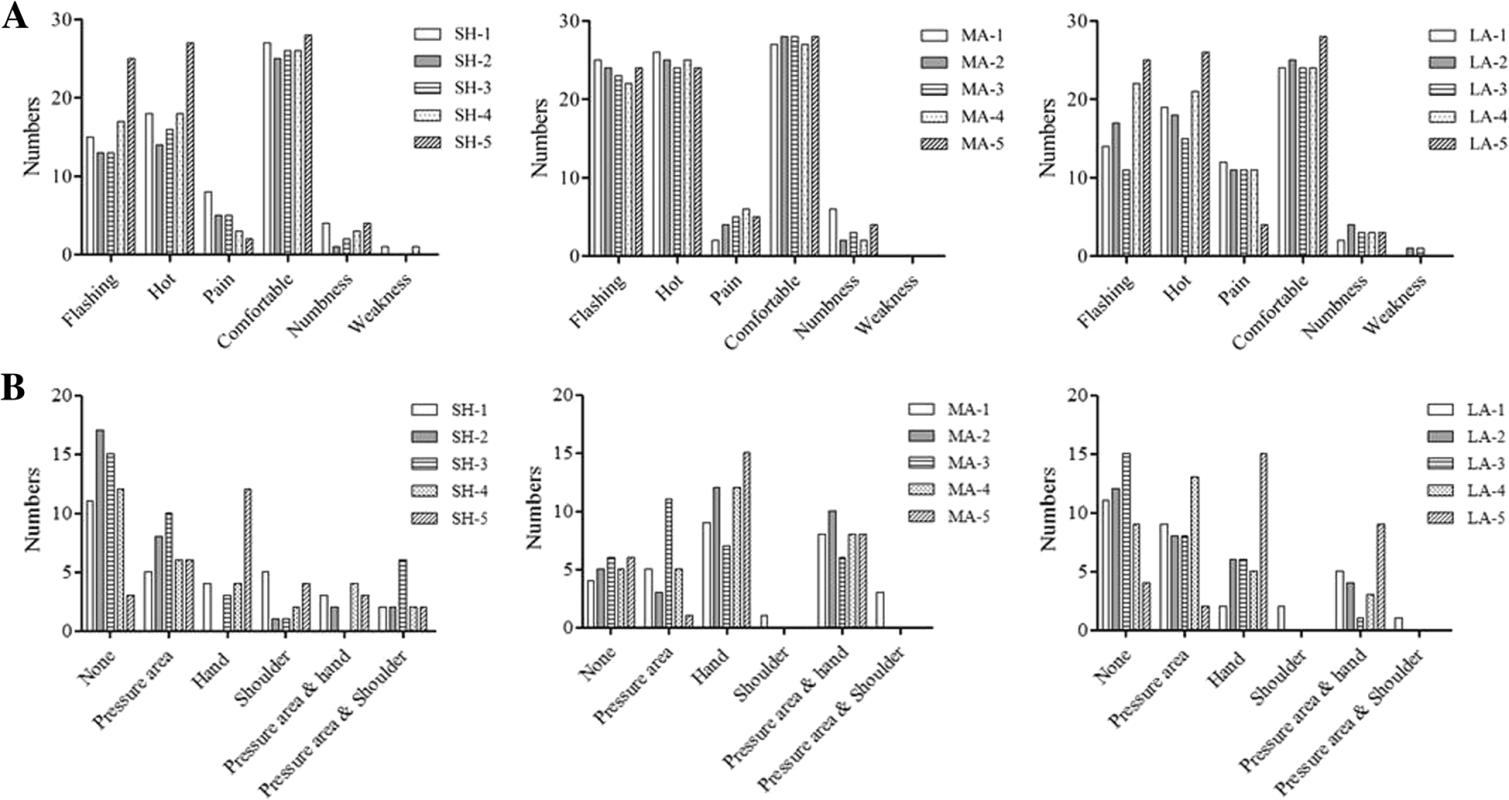
**a** The perceptive of participants after pressure application **b** Hot flushes feeling area at MaSPs from the questionnaires of BF and ST of each timepoint: time 1 = 0 s, 2 = 30 s, 3 = 60 s, 4 = 180 s, and 5 = 300 s

In Fig. 8a, all participants, 85 % report a sensation of the hot flushed of 8 points: MaSP-SH-5, MaSP-MA-1 to 5, MaSP-LA-4 and MaSP-LA-5. The comfortable was the most feelings of all points (80–93.3 %). All MaSPs are associated with numbness, but in less than 20 % of participants. Less than 5 % of participants report weakness of arm when pressure is applied to MaSP-SH-1, MaSP-SH-4, MaSP-LA-2 and MaSP-LA-3. In Fig. 8b, more than 40 % of the MaSPs of shoulder are associated with the sensation of the hot flushes which primarily involved the pressure area and radiating to the hand, and to a lesser extent the shoulder. The MaSPs of lateral side of the arm are associated with the sensation of the hot flushes which primarily involved the pressure area and radiating to the hand. Conversely, the MaSPs of medial side of the arm are associated with the sensation of the hot flushes involving the hand more than the pressure area.

## Discussion

This is the first of MaSPs on anatomical correlations and physiological effects on BF and ST on neck, shoulder, and arm areas. The first goal of this study was to investigate anatomical surface landmarks and structures of 15 MaSPs on the neck, shoulder, and arm using cadaveric subjects. In this study, surface landmarks described for all MaSPs are similarly to that being described in tradition CTTM textbooks [1, 5] and clinical practice. Therefore, surface landmarks of this study might be an evidence confirming location of MaSPs in human.

MaSPs are found to be mainly muscle structures with associated their vessels and nerve branches. In previous studies, massage treatments were shown to promote relieve of musculoskeletal symptoms such as decreased muscle tension or spasm, reduced pain, decreased stiffness of joint [6, 15, 16], and increased blood circulation and relaxation [11]. Thus, pressure application to MaSPs relates to the mechanics of massage pressure which passes through the muscles [17]. The possible mechanism of reducing muscular pain may be explained by gate control theory [18] or release of neurotransmitters such as beta-endorphin after massage [19]. Decreased muscle tension may be explained by increasing of the length of muscle fiber after massage [20]. Moreover, this study confirmed that MaSP-SH-2 to 4 were anatomically separate from the location of the common carotid arteries. Massage on carotid artery may lead to syncope [21, 22] and affect blood circulation particularly in brain. Thus, this study also confirmed the safety of massage on the neck area when applied appropriately.

The second goal of this study was investigate physiological effects of massage on In BF and ST. In BF study, only 7 MaSPs out of 15 MaSPs were selected because in our preliminary study, the others MaSPs showed no change. The briefness of the changes in BF at each MaSP can be accounted for by physiological autoregulation [23]. Moreover, in clinical practice of CTTM, massage treatment combined both basic massage and MaSPs, for a long treatment duration of 45 min [1, 4, 5]. Thus, the effect on blood circulation can be longer-lasting. This phenomenon may be similar to that of reactive hyperemia [24], although no net BF measurements were done. CTTM described a maneuver where pressure is applied to specific MaSP such as MaSP-MA-2 which is located above brachial artery. This is believed to open the wind gate or Perd-Pra-Tu-lom in Thai, for the purpose of increasing perfusion to that area. However, further studies are suggested to clarify this effect.

Several previous studies have suggested (without providing directly measured data) that massage may increase the rate of BF and improving blood circulation [25, 26]. On the other hand, previous studies which directly measured arterial blood flow demonstrated BF decreased or did not change during or after massage [27–29]. Mechanical pressure from massage has been suggested to affect superficial arteries as well as deep arteries [9]. Our study confirms a small increase in BF after pressure application as well as the perception of hot flushes through the hand area. Thus, pressure application MaSPs can be associated with minor short term changes in BF both at the area and distal to it. Further studies are suggested to identify change of BF after all of the MaSPs and after massage treatment session.

MaSPs resulted in increasing ST which can signify increasing blood circulation [11]. The lack of changes in ST at other measurement points can be accounted for by the fact that the duration of pressure applied to each MaSP was brief and the body’s thermoregulation [30] overcame this effect. Moreover, the effects of massages are different based on various factors such as location, duration, posture, and massage procedure.

Even though this study failed to establish a relationship between BF and ST, the scatter plots did demonstrate a positive trend. In this study, BF measurement varies depending upon individuals and might be easily interfered by emotions and environments.

The perception from questionnaires showed that most participants feel comfortable after pressure application, and reported minimally pain and weakness. The weakness reported can be due to fixed posture of the right arm during the experiment. However, these effects disappeared after 10 min of resting. Thus, massages on MaSPs results in relaxation without serious complications.

Although we excluded some cadavers with fixed posture from our study, the fixed position of cadavers still presented a major limitation of anatomical study especially in shoulder and axillary areas. This study design may be more suited to soft cadavers. The measurements of BF and ST on different days presented another limitation. Because of its effect on the different responses that could be interfered by different surroundings. However, we tried to overcome such effect by taking measurements at the same time of day and in the same setting. The Duplex ultrasound also had its own limitation. It could not measure microcirculation. We believe that some small arterioles may change after massage all MaSPs. However, this is the best machine that we can find in this study. Therefore, further studies are needed to investigate microcirculation change after massage by using proper machine such as a Sensor Photoplethysmography.

CTTM is being used for treating musculoskeletal diseases globally, however understanding of anatomical structures and its relations of each massage point are needed. This study demonstrates its safety, as well as the information to be used as cautions, and contraindications when press on MaSPs of the neck, shoulder, and arm areas in clinical practice. Finally, based on anatomical structures of MaSPs and physiological effects of massage, the mechanism of massage and its effects in many diseases can be proposed and studied further.

## Conclusions

The anatomical surface landmarks and structures of MaSPs on the neck, shoulder and arm have been revealed in this study. These help identify structures and distinguish the benefits and responses of each MaSP. Importantly, this study showed that massage on mostly MaSPs were not directly on the large arteries and nerves. MaSPs can cause significant, but brief, increases in BF and ST CTTM can be sued without complications.

## Abbreviations

BF: Blood flow
CTTM: Court-type Thai traditional massage
DA: Diameter of artery
LA: Lateral side of the arm
MA: Medial side of the arm
MaSP: Major signal point
PS: Peak systolic velocity
SH: Shoulder
ST: Skin temperature
